# Comparison between bacterial bio-formulations and gibberellic acid effects on *Stevia rebaudiana* growth and production of steviol glycosides through regulating their encoding genes

**DOI:** 10.1038/s41598-024-73470-0

**Published:** 2024-10-15

**Authors:** Amal M. Abdelsattar, Mohamed A. El-Esawi, Ashraf Elsayed, Yasmin M. Heikal

**Affiliations:** 1 Botany Department, Faculty of Science, Mansoura University, Mansoura, 35516 Egypt; 2 Botany Department, Faculty of Science, Tanta University, Tanta, 31527 Egypt

**Keywords:** *Stevia rebaudiana*, Gibberellic acid, Plant growth promoting bacteria, Endophytic bacteria, *Azospirillum brasilense*, UDP glycosyltransferases (UGTs)., Microbiology, Molecular biology, Plant sciences

## Abstract

*Stevia rebaudiana* is associated with the production of calorie-free steviol glycosides (SGs) sweetener, receiving worldwide interest as a sugar substitute for people with metabolic disorders. The aim of this investigation is to show the promising role of endophytic bacterial strains isolated from *Stevia rebaudiana* Egy1 leaves as a biofertilizer integrated with *Azospirillum brasilense* ATCC 29,145 and gibberellic acid (GA_3_) to improve another variety of stevia (*S. rebaudiana* Shou-2) growth, bioactive compound production, expression of SGs involved genes, and stevioside content. Endophytic bacteria isolated from *S. rebaudiana* Egy1 leaves were molecularly identified and assessed in vitro for plant growth promoting (PGP) traits. Isolated strains *Bacillus licheniformis* SrAM2, *Bacillus paralicheniformis* SrAM3 and *Bacillus paramycoides* SrAM4 with accession numbers MT066091, MW042693 and MT066092, respectively, induced notable variations in the majority of PGP traits production. *B. licheniformis* SrAM2 revealed the most phytohormones and hydrogen cyanide (HCN) production, while *B. paralicheniformis* SrAM3 was the most in exopolysaccharides (EPS) and ammonia production 290.96 ± 10.08 mg/l and 88.92 ± 2.96 mg/ml, respectively. Treated plants significantly increased in performance, and the dual treatment T7 (*B. paramycoides* SrAM4 + *A. brasilense*) exhibited the highest improvement in shoot and root length by 200% and 146.7%, respectively. On the other hand, T11 (*Bacillus cereus* SrAM1 *+ B. licheniformis* SrAM2 + *B. paralicheniformis* SrAM3 + *B. paramycoides* SrAM4 + *A. brasilense* + GA_3_) showed the most elevation in number of leaves, total soluble sugars (TSS), and up-regulation in the expression of the four genes *ent-KO*, *UGT85C2*, *UGT74G1* and *UGT76G1* at 2.7, 3.3, 3.4 and 3.7, respectively. In High-Performance Liquid Chromatography (HPLC) analysis, stevioside content showed a progressive increase in all tested samples but the maximum was exhibited by dual and co-inoculations at 264.37% and 289.05%, respectively. It has been concluded that the PGP endophytes associated with *S. rebaudiana* leaves improved growth and SGs production, implying the usability of these strains as prospective tools to improve important crop production individually or in consortium.

**Supplementary Information:**

The online version contains supplementary material available at 10.1038/s41598-024-73470-0.

## Introduction

Agriculture is the most critical resource for the worldwide market and environmental systems face several challenges. The global consumption of chemical fertilizers has increased and played a vital role in crop yields^1^. However, plants grown in this way do not have enough time to grow and mature properly, and therefore do not acquire excellent plant attributes^2^. Moreover, highly harmful toxic compounds will build up in the human body and soil^3^. Thus, developing eco-friendly and sustainable solutions that could reduce the need for chemical fertilizers is a demand. Beneficial microbes could be a significant source of bioactive natural compounds^4^.

Plant growth-promoting bacteria (PGPB) have three main functions: producing essential metabolites for plants, enabling the uptake of nutrients from the soil, and reducing disease in plants^5,6^. The majority of investigations have focused on bacteria found in the plant rhizosphere, and one of them is plant growth-promoting rhizobacteria (PGPR)^7^. However, a subgroup of bacteria from the rhizosphere can enter and colonize plants as endophytes, creating a mutualistic relationship with the plants without harmful effect^8^. Endophytes can promote plant growth directly through phosphate solubilization, nitrogen fixation^9^, and phytohormone production such as indole-3-acetic acid (IAA) and gibberellic acid (GA)^10^, and indirectly through exopolysaccharide (EPS) production^11^and inhibiting phytopathogens by hydrogen cyanide (HCN) and hydrolyzing enzymes^12^.

Endophytic communities in leaves vary depending on the host plant species or genotype, plant growth stage, and plant morphology^13^. Several studies have reported that the genera *Azospirillum*, *Bacillus*, *Azotobacter*, *Pseudomonas*, and *Enterobacter *have the potential to promote plant growth^14^. Furthermore, *Bacillus* and *Azospirillum *are the most well-studied genera of PGPB, improving plant growth and yield^15^. Co-inoculation of legumes with *Azospirillum brasilense* and *Bradyrhizobium *has been shown to boost grain yield in maize, wheat, soybean and common bean^16,17^. It has been demonstrated that inoculating *Salicornia bigelovii* with *Bacillus licheniformis *and other PGPB can work together to improve plant growth and nutrient uptake^18^. *A. brasilense* Sp7 and *Bacillus spharecus *UPMB10 improved root growth in dessert-type banana^19^. Similar to this, fennel seeds struggle to establish themselves due to their poor vegetative growth and poor seedling emergence, particularly in drought-stressed environments^20^. By boosting antioxidant activity and defense mechanisms, seed inoculation treatments with *Pseudomonas fluorescens* bacteria and *Trichoderma harzianum *fungus improved the early growth of fennel seedlings under drought-stress conditions^21^. In *Stevia*, research has been conducted to assess the impact of mycorrhizal fungi and plant growth-promoting rhizobacteria (PGPR) on the organism’s ability to grow, accumulate secondary metabolites, and express genes involved in biosynthesis. According to studies by Rahi, et al.^22^ inoculating plants with distinct PGPR enhanced their growth, photosynthetic indices, and stevioside and rebaudioside A accumulation. On the other hand, inoculation of *Stevia* with plant-growth-promoting rhizobacteria and arbuscular mycorrhizal fungus stimulate plant height, stevioside, minerals, and pigments content^23^. Oviedo-Pereira, et al.^24^ reported that the stimulating endophytes *Enterobacter hormaechei* H2A3 and H5A2 increased the SG content, stimulated the density of trichomes in the leaves, and encouraged the accumulation of specialized metabolites in trichomes; nevertheless they did not promote plant growth.

Egypt’s economy is challenged by a shortfall of 0.843 million tons between sugar supply and demand^25^. Accordingly, alternative biotechnology approaches can be employed in the large-scale cultivation of perennial shrub *Stevia rebaudiana*, which has been utilized as a non-caloric natural sweetener for those with metabolic disorders^26^. Its leaves contain steviol glycosides (SGs), a class of tetracyclic diterpenoid compounds estimated to be 300 times sweeter than sucrose^27^.

Stevioside and rebaudioside A are the two main sweetening components, while rebaudioside C, D, E, and F, dulcoside A, and steviolbioside are minor^28^. SGs are produced in the chloroplast via the non-mevalonate pathway of methyl-erythritol phosphate (MEP)^27^. The structures of both SGs and GA are tetracyclic diterpenes and share the same biosynthetic pathway derived from the synthesis of ent-kaurenoic acid^29^. Several glycosylation reactions catalyzed by UDP glycosyltransferases (UGTs) make up the biosynthetic pathway for different SGs^30^.

GA_3 _is one of the most essential and bioactive gibberellin-like proteins^31^. Exogenous GA_3_ treatment in *S. rebaudiana*has increased stem elongation and flowering^32^. On the other side, several studies reported that GA_3 _as an elicitor which stimulates secondary metabolite production in plants such as antioxidants and pigments as well as a defense mechanism^33^.

Sequels to these different microbe bio-formulations with *A. brasilense* and GA_3_ were designed to test and compare their effects on *S. rebaudiana* Shou-2 plant growth performance, bioactive compounds such as stevioside production, and up-regulation of the genes responsible for the SGs pathway (Table 1).

**Table 1:** Design of treatments.

Code	Treatments	Code	Treatments
T0	Control	T6	*A.brasilense* + *B.paralicheniformis* SrAM3
T1	*Bacillus licheniformis* SrAM2	T7	*A.brasilense* + *B.paramycoids* SrAM4
T2	*Bacillus paralicheniformis* SrAM3	T8	*A.brasilense* + GA_3_
T3	*Bacillus paramycoides* SrAM4	T9	*B. cereus* SrAM1 + *B. licheniformis* SrAM2 + *B. paralicheniformis* SrAM3 + *B. paramycoides* SrAM4
T4	Gibberellic acid (GA_3_)	T10	*B. cereus* SrAM1 + *B. licheniformis* SrAM2 + *B. paralicheniformis* SrAM3 + *B. paramycoides* SrAM4 + *A. brasilense*
T5	*Azospirillum brasilense* + *B. licheniformis* SrAM2	T11	*B. cereus* SrAM1 *+ B. licheniformis* SrAM2 + *B. paralicheniformis* SrAM3 + *B. paramycoides* SrAM4 + *A. brasilense* + GA_3_

## Results

### Isolation, selection, and identification of endophytic bacteria

**Fig. 1:**
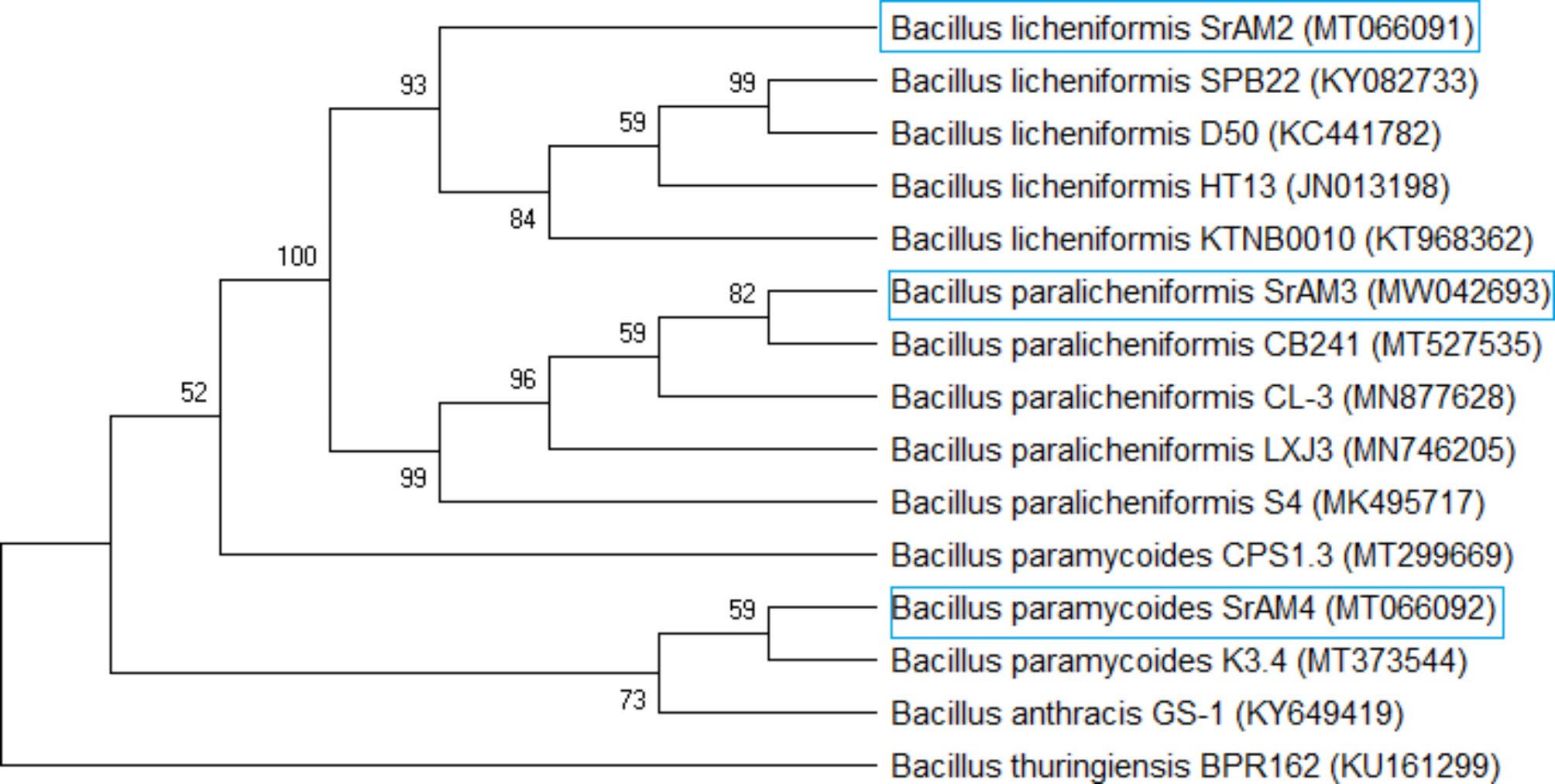
Phylogenetic relationship of isolated strains relative to other type strains. GenBank accession numbers are indicated in parentheses.

Three strains from *S. rebaudiana* Egy1 leaves were isolated, purified, colonies described, and given SrAM2, SrAM3, and SrAM4 codes. In SrAM2, the colonies were buff powdery flat punctiform with irregular and filamentous margins. SrAM3 exhibited white powdery raised large colonies with irregular and lobate margins, while SrAM4 revealed creamy convex moderate colonies with round and entire round margins. All of the bacteria were gram-positive and formed spores. Isolates were molecularly identified as *Bacillus licheniformis* SrAM2 (100%), *Bacillus paralicheniformis* SrAM3 (100%), and *Bacillus paramycoides* SrAM4 (99.79%) with accession numbers MT066091, MW042693, and MT066092, respectively, based on sequence identity (Fig. 1).

### Plant growth-promoting characteristics of endophytic bacteria

It can be observed that all strains produce a high amount of GA_3_ compared with IAA. Still, the elevated IAA and GA_3_ syntheses were conducted by *B. licheniformis* SrAM2 at 52.97 and 186.23 µg/mL, respectively (Table 2). The potential of bacteria to fix nitrogen is indicated by their growth on a nitrogen-free medium, and all strains were able to do so. Positive P solubilization, on the other hand, revealed clear zones around the colonies of *B. licheniformis* SrAM2 and *B. paralicheniformis* SrAM3, but no clear zones surrounding *B. paramycoides* SrAM4 colonies. All strains produced EPS and ammonia, but *B. paralicheniformis* SrAM3 displayed the maximum production. HCN production was observed in *B. licheniformis* SrAM2 864.86 mg/L, whereas *B. paralicheniformis* SrAM3 and *B. paramycoides* SrAM4 were not detected, as represented in Table 2.

**Table 2:** Determination of phenotypic characterization of in vitro plant growth-promoting criteria of isolated strains.

Plant growth-promoting traits (PGP)	*B. licheniformis* SrAM2	*B. paralicheniformis* SrAM3	*B. paramycoides* SrAM4
Quantitative Characterization			
Indole acetic acid (IAA) (mg/mL)	52.97 ± 1.03^a^	17.46 ± 4.77^b^	31.51 ± 6.14^b^
Gibberellic acid (GA) (mg/mL)	186.23 ± 6.45^a^	167.15± 3.86^b^	133.92 ± 3.09^c^
Exopolysacchrides (EPS) (mg/L)	169.07 ± 3.91^b^	290.96 ± 10.08^a^	131.36 ± 3.79^c^
Ammonia production (mg/mL)	62.32 ± 2.15^b^	88.92 ± 2.96^a^	49.47 ± 4.94^c^
Hydrogen cyanide (HCN) (mg/L)	864.86 ± 29.96^a^	-	-
Qualitative Characterization			
Nitrogen fixation	+	+	+
Phosphate Solubilization	+	+	-
Amylase	-	+	+
Cellulase	+	-	+
Protease	-	+	+
Lipase	-	+	+

The results were recorded as mean of triplicates ± (SE). Different superscript letters refer to significant differences (*P* ≤ 0.05).

### Extracellular hydrolytic enzymes activity

Amylase, cellulase, and protease activity were detected as clear zone surrounding a colony, whereas a white precipitation around colonies indicated lipase activity. As illustrated in Table 2, B. *paramycoides* SrAM4 could produce all hydrolytic enzymes, but *B. paralicheniformis* SrMA3 had the ability to produce of all enzymes except cellulase, whereas *B. licheniformis* SrAM2 produced cellulase only.

### Effect of treatments on growth parameters

**Fig. 2:**
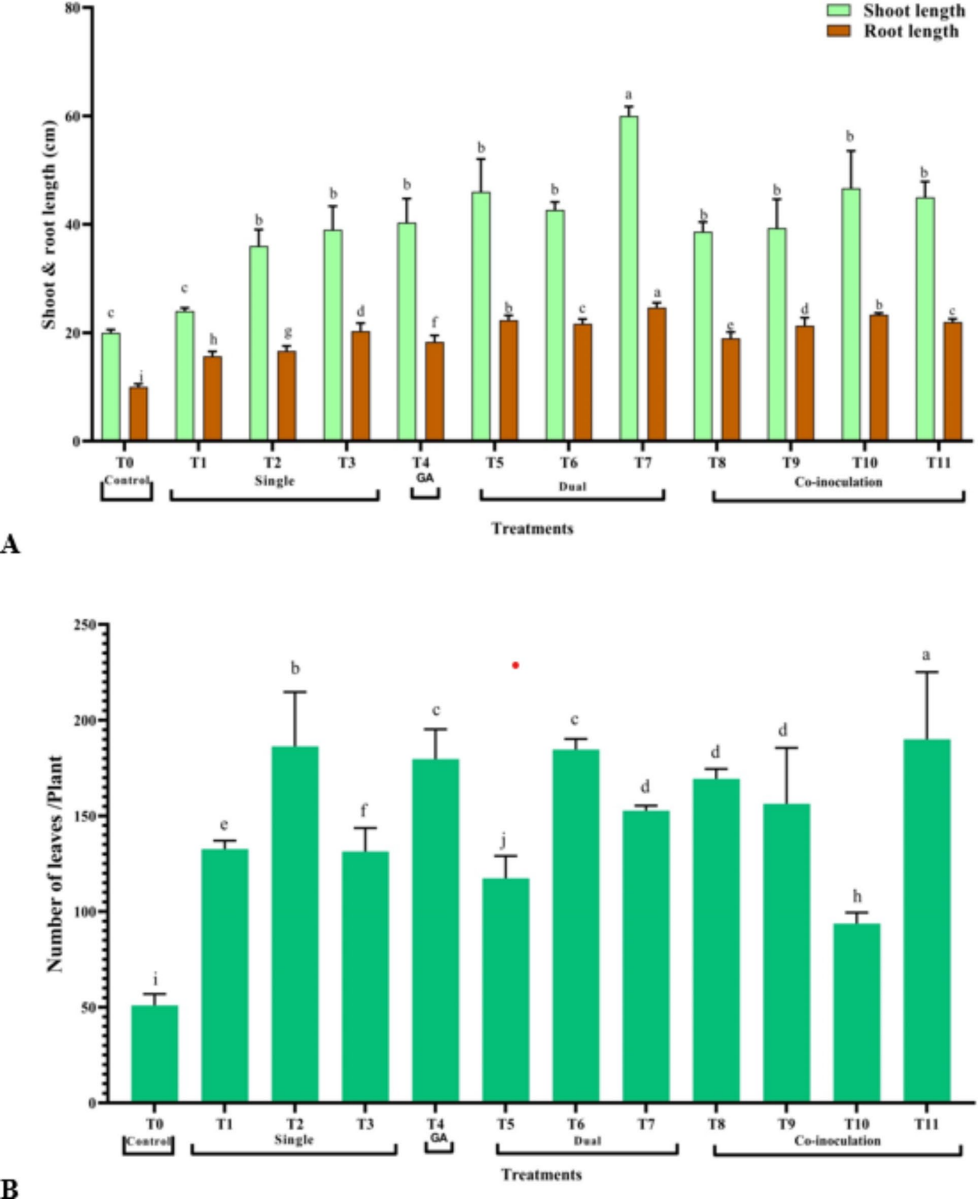
Effect of treatments on morphological parameters of *S. rebaudiana* Shou-2 plantlets **(A)** Shoot and root length and **(B)** Number of leaves. Bar indicated ± SE. Different lower-case letters indicated significant difference at *P* ≤ 0.05 [Duncan’s test].

Results showed that plants treated with dual inoculations showed higher growth parameters than those with individual and co-culture treatments. The maximum increase in plant growth was observed in the dual treatment T7, resulting in an increase of 200% and 146.7% in shoot and root length, respectively (Fig. 2A). Number of leaves showed the maximum in co-culture treatment T11, followed by T2, with an increase of 272.55% and 264.71%, respectively (Fig. 2B).

### Effect of treatments on pigment content

The highest chl a and chl b, carotenoids, and total pigment content were observed in plants treated with individual treatments as illustrated in Table 3, but the maximum chl a and chl b and total pigment content were achieved by T4 at 2.43, 2.88, and 5.97 mg/g f.wt., respectively. Carotenoids content of 0.79 mg/g f.wt. was the maximum in T3.

**Table 3:** Effect of treatments on chl a, chl b, carotenoids, and total pigments of *S. Rebaudiana* Shou-2.

Treatments	Chlorophyll a (mg/g)	Chlorophyll b (mg/g)	Carotenoids (mg/g)	Total pigment (mg/g)
T0	0.44 ± 0.02^k^	0.63 ± 0.04^f^	0.35 ± 0.07^g^	1.43 ± 0.05^k^
T1	1.70 ± 0.02^b^	1.07 ± 0.03^c^	0.75 ± 0.00^b^	3.51 ± 0.04^b^
T2	1.31 ± 0.06^e^	0.87 ± 0.16^d^	0.52 ± 0.06^e^	2.70 ± 0.18^e^
T3	1.59 ± 0.01^c^	0.61 ± 0.01^f^	0.79 ± 0.00^a^	2.99 ± 0.01^c^
T4	2.43 ± 0.10^a^	2.88 ± 0.22^a^	0.65 ± 0.03^c^	5.97 ± 0.29^a^
T5	1.41 ± 0.03^d^	1.17 ± 0.02^b^	0.51 ± 0.03^e^	3.08 ± 0.03^c^
T6	0.98 ± 0.07^i^	0.60 ± 0.25^f^	0.48 ± 0.17^f^	2.06 ± 0.18^i^
T7	1.41 ± 0.05^d^	0.87 ± 0.17^d^	0.61 ± 0.11^d^	2.89 ± 0.02^d^
T8	0.86 ± 0.01^j^	0.42 ± 0.07^g^	0.44 ± 0.03^f^	1.73 ± 0.03^j^
T9	1.02 ± 0.07^h^	0.74 ± 0.01^e^	0.49 ± 0.02^e^	2.25 ± 0.05^h^
T10	1.17 ± 0.20^g^	0.87 ± 0.16^d^	0.49 ± 0.13^e^	2.54 ± 0.16^f^
T11	1.22 ± 0.02^f^	0.59 ± 0.07^f^	0.61 ± 0.06^d^	2.42 ± 0.03^g^

The results were recorded as mean of triplicates ± standard error (SE). Different superscript letters refer to significant differences (*P* ≤ 0.05) (Duncan’s multiple range test).

### Effect of treatments on total soluble sugars, carbohydrates, and protein

**Fig. 3:**
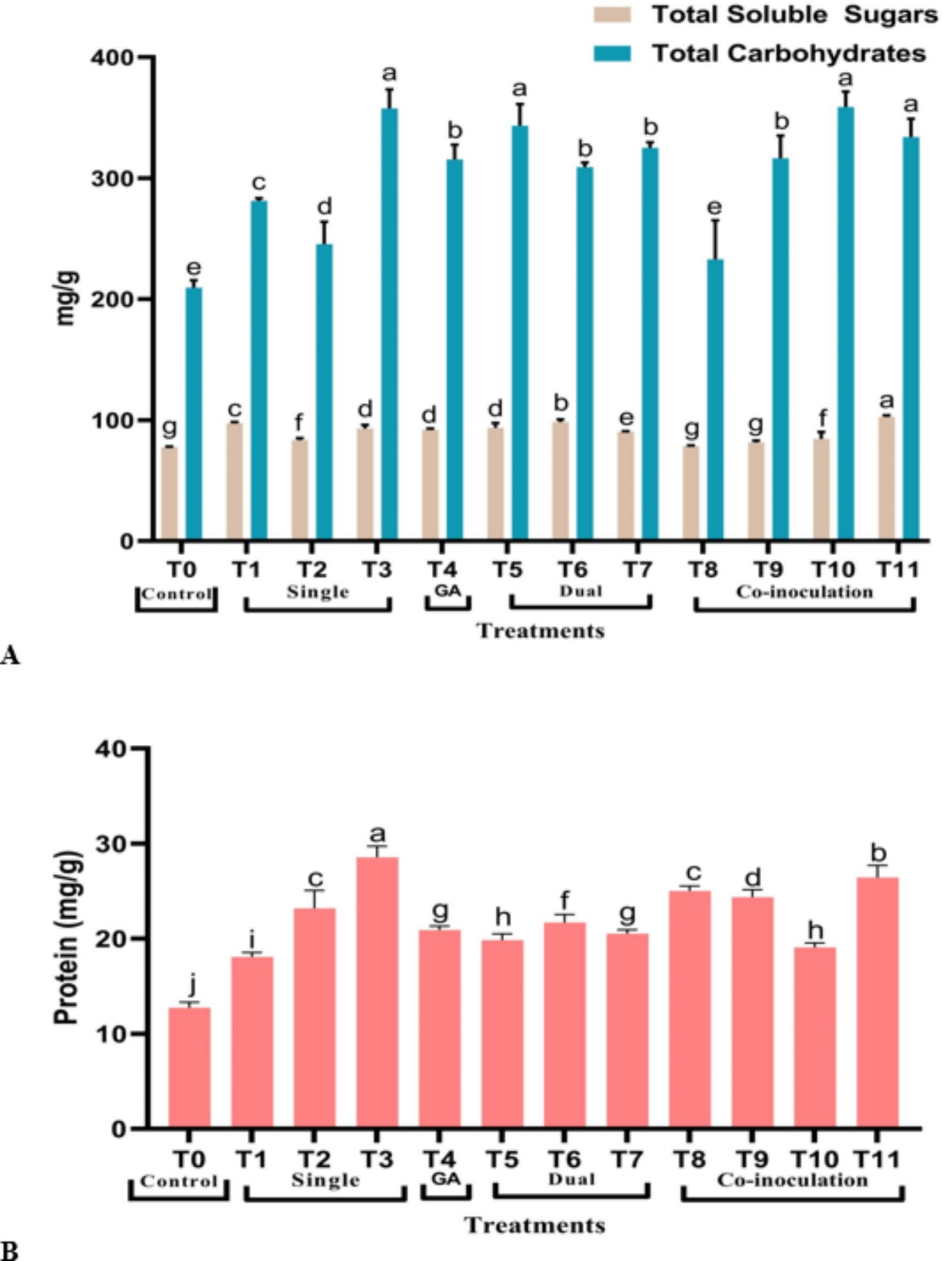
Variation in biochemical parameters in *S. rebaudiana* Shou-2 **(A)** TSS, total carbohydrates contents in dry leaves and **(B)** Total protein content in fresh leaves. Bar indicated ± SE. Different lower-case letters indicated significant difference at *P* ≤ 0.05 [Duncan’s test].

Compared to the control, all treatments showed elevations in TSS, total carbohydrates (Fig. 3A), and protein (Fig. 3B). The treatment T11 showed the maximum content of total soluble sugars at 102.56 mg/g d.wt. On the contrary, total carbohydrates represented the highest by T10 (358.96) mg/g d.wt, whereas T3 outlined the maximum protein content at 28.55 mg/g f.wt.

### Effect of treatment on non-enzymatic antioxidants

In Table 4, T5 revealed highest TPC content at 55.34 mg (GAEs)/g. TFC was the highest by T4 at 9.63 mg (QE)/g. The DPPH radical scavenging activity was significantly reduced by T11 (55.24%). Additionally, maximum TAC was induced by T2 at 34.55 mg (AA)/g.

**Table 4:** Effect of treatments on non-enzymatic antioxidants activity of *S. Rebaudiana* Shou-2.

Treatments	Total phenolics (mg (GAEs)/g)	Total flavonoids (mg (QE)/g)	Total antioxidant capacity (mg (A.A)/g)	DPPH radical scavenging activity (%)
T0	21.72 ± 0.72^j^	4.53 ± 0.05^h^	8.05 ± 0.22^h^	78.12 ± 1.31^a^
T1	30.13 ± 0.84^h^	5.96 ± 0.14^g^	17.39 ± 0.49^f^	77.17 ± 1.78^b^
T2	34.03 ± 0.95^f^	7.07 ± 0.16^f^	34.55 ± 3.34^a^	71.55 ± 1.18^c^
T3	30.30 ± 0.92^h^	5.97 ± 0.10^g^	26.84 ± 4.51^b^	71.83 ± 2.13^c^
T4	39.47 ± 1.23^b^	9.63 ± 0.10^a^	21.35 ± 1.04^d^	66.66 ± 1.97^e^
T5	55.34 ± 1.60^a^	6.65 ± 0.23^f^	19.05 ± 0.26^e^	61.64 ± 2.20^f^
T6	37.04 ± 1.23^d^	8.79 ± 0.34^d^	17.74 ± 0.85^e^	67.27 ± 2.40^e^
T7	34.68 ± 1.08^e^	9.35 ± 0.12^b^	16.76 ± 1.48^f^	70.80 ± 2.10^d^
T8	30.00 ± 1.28^h^	5.83 ± 0.05^g^	13.39 ± 0.64^g^	61.38 ± 2.19^f^
T9	33.01 ± 1.03^g^	7.61 ± 0.15^e^	23.88 ± 2.24^c^	66.21 ± 1.10^e^
T10	29.19 ± 0.90^i^	5.92 ± 0.13^g^	20.96 ± 0.97^d^	69.57 ± 2.48^d^
T11	37.87 ± 1.27^c^	8.87 ± 0.24^c^	21.35 ± 0.63^d^	55.24 ± 1.97^g^

The results were recorded as mean of triplicates ± standard error (SE). Different superscript letters refer to significant differences (*P* ≤ 0.05) (Duncan’s multiple range test).

### Effect of treatments on enzymatic antioxidants

**Fig. 4:**
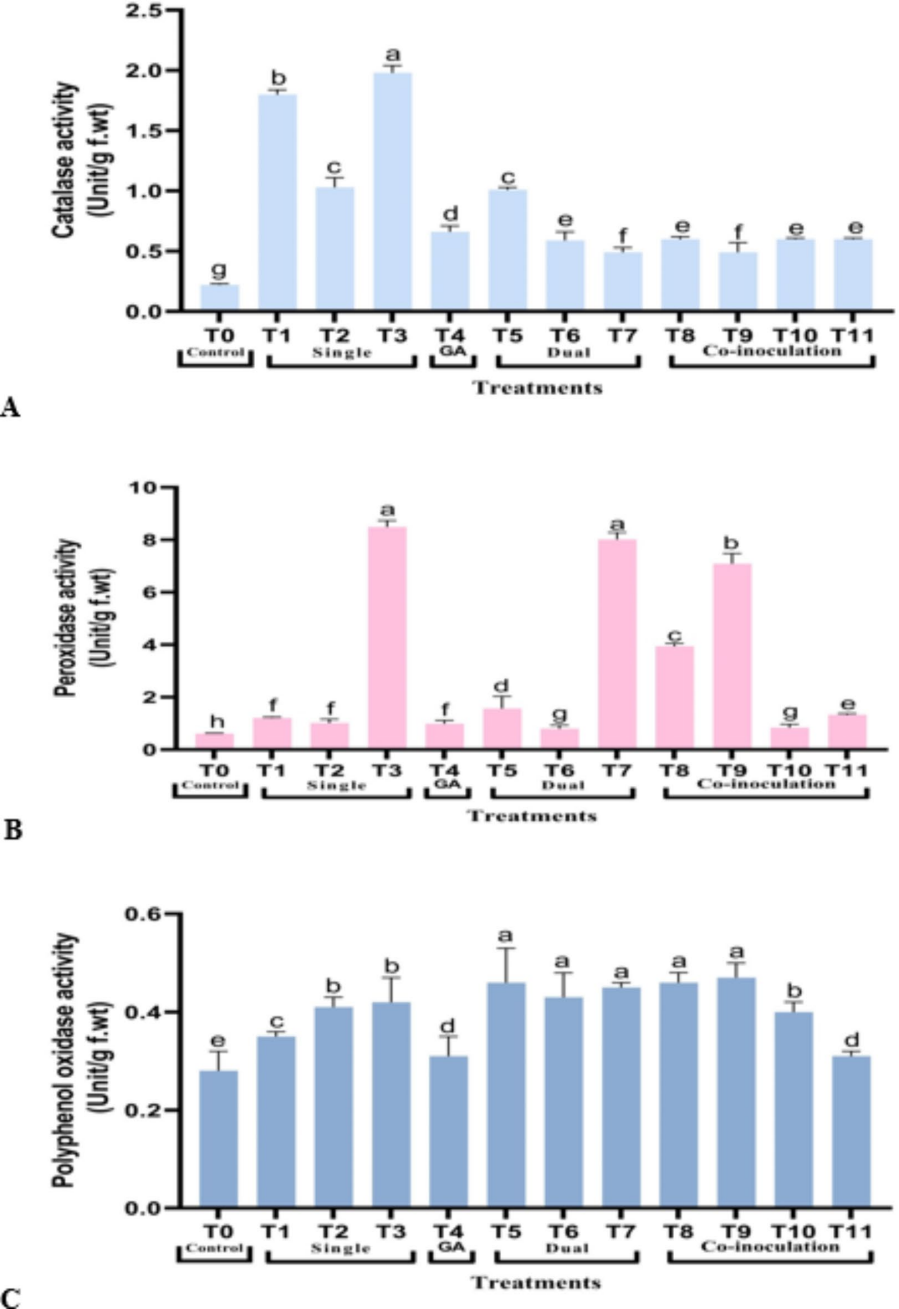
Changes in the activities of **(A)** Catalase, **(B)** Peroxidase, and **(C)** Polyphenol oxidase of *S. rebaudiana* Shou-2 leaves expressed as Unit/g f.wt. Bar indicated ± SE. Different lower-case letters indicated significant difference at *P* ≤ 0.05 [Duncan’s test].

As shown in Fig. 4, all enzymatic antioxidant activities were increased compared to control. For example, treatment T3 showed the highest CAT and POD activities, 1.98 and 8.49 Unit/g f.wt., respectively. Conversely, PPO activity showed the highest at T9 (0.47 Unit/g f.wt.).

### Effects of treatments on the transcription of SGs-related genes

**Fig. 5:**
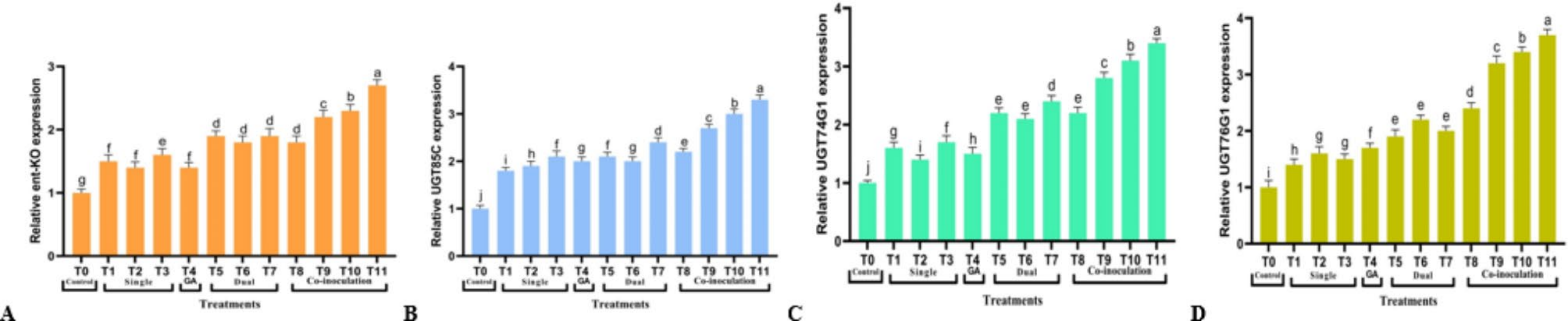
The genes expression levels of *S. rebaudiana* Shou-2 plantlets **(A)** ent-KO, **(B)** UGT85C, **(C)** UGT74G1 and **(D)** UGT76G1. Bar indicated ± SE. Different lower-case letters indicated significant difference at *P* ≤ 0.05 [Duncan’s test].

The evaluation of transcription patterns in the regulatory SGs-related genes under the effect of treatments is shown in Fig. 5; the results illustrated that all applications up-regulated the transcription of the SGs genes relative to control, varying with different treatments. Various treatments significantly impacted *ent-KO*, *UGT85C2*, *UGT74G1*, and *UGT76G1* transcription. Still, the treatment T11 had the highest relative gene expression at 2.7, 3.3, 3.4, and 3.7, respectively.

### Effect of treatments on stevioside content by HPLC analysis

**Fig. 6:**
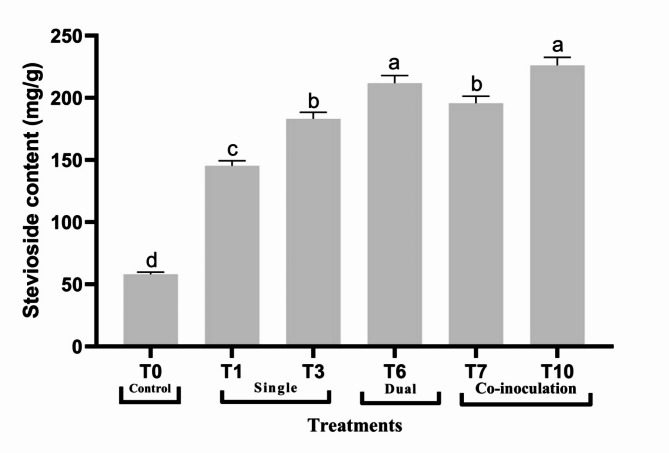
Effect of some treatments on stevioside content, selected according to the elevation in some biochemical attributes. Bar indicated ± SE. Different lower-case letters indicated significant difference at *P* ≤ 0.05 [Duncan’s test].

As illustrated in Fig. 6, stevioside analysis of some selective samples was performed by HPLC using different concentrations of the standard solution illustrated in Supplementary Fig. 1. The maximum stevioside content at 226.09 mg/g, as shown in the chromatogram was represented by T10, with an increase of 3.9 times more than the minimum concentration (control) at 58.11 mg/g. (Supplementary Fig. 2).

### Intercorrelation between different treatments

**Fig. 7:**
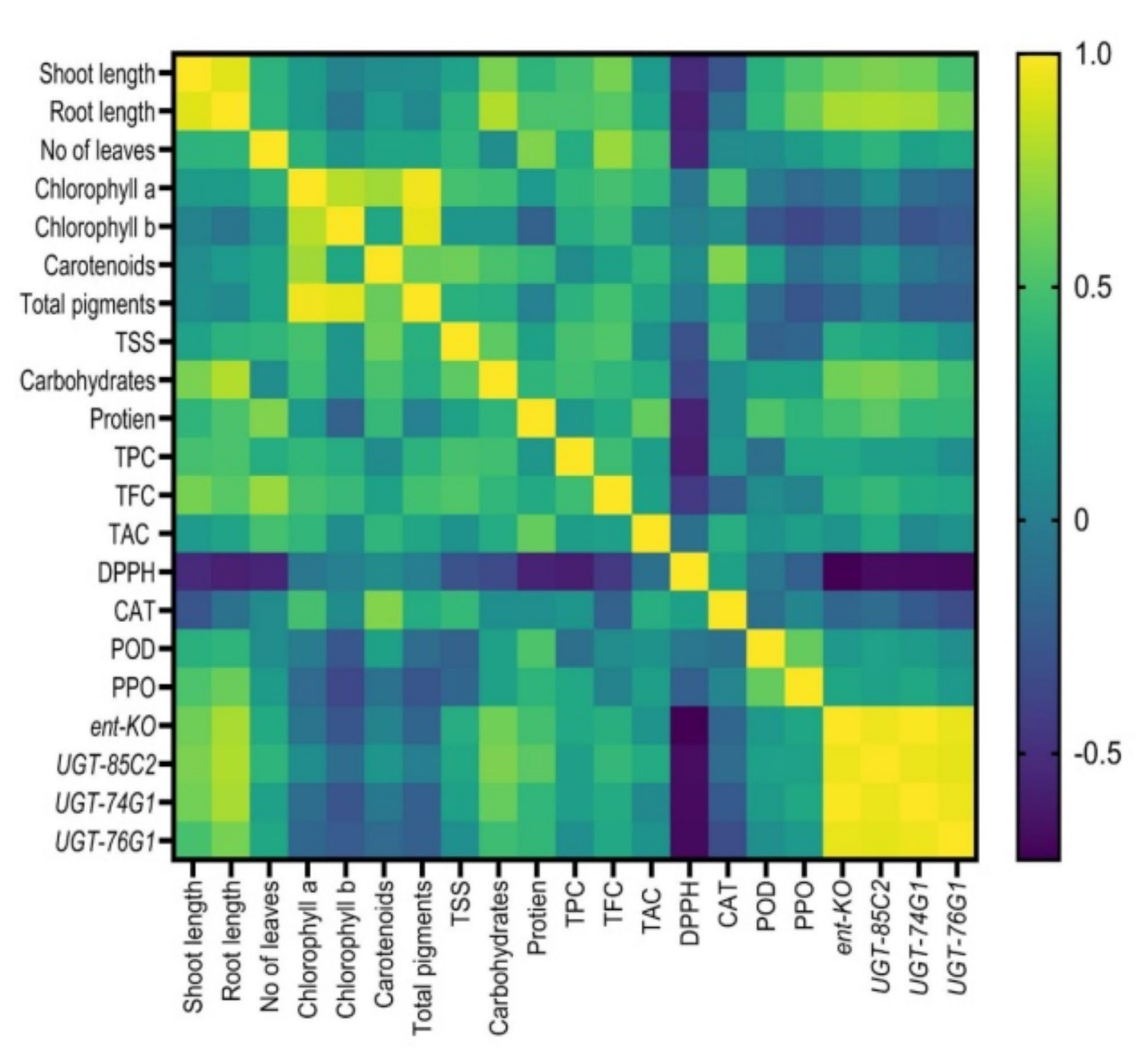
Heatmap of the simple linear Pearson correlation coefficient between growth parameters, biochemical attributes, and the expression of genes involved in steviol glycosides production. The colours reflect significant correlation values in each treatment, yellow for high intensities and blue for low intensities (follow scale at right).

Pearson’s test was used to assess the possible correlation between morphological and biochemical parameters and gene transcription levels (Fig. 7 and Supplementary Table 2). Shoot length (SHL) had a significantly positive correlation with root length (RL), carbohydrates content, TFC, and the expression of *ent-KO*, *UGT85C2*, and *UGT74G1* genes (0.923, 0.655, 0.641, 0.620, 0.659, and 0.631, respectively). In contrast, SHL was negatively correlated with DPPH and CAT by − 0.525 and − 0.275, respectively. Similarly, RL was significantly positively correlated with carbohydrates content, PPO, and the four transcribed genes 0.801, 0.608, 0.779, 0.791, 0.774, and 0.644, respectively. Conversely, RL negatively correlated with chlb, DPPH, and CAT at − 0.056, − 0.575, and − 0.090, respectively. Correspondingly, the number of leaves was significantly positively correlated with protein and TFC (0.674 and 0.737, respectively), whereas it was negatively correlated with DPPH (− 0.547). In the same manner, total pigments were positive with chla, chlb, and carotenoids (0.964, 0.938, and 0.599, respectively). In addition, carotenoids were significantly positive correlated with TSS and CAT (0.617 and 0.677, respectively). Carbohydrates significantly positively correlated with the expression of three relative genes (*ent-KO*, *UGT85C2*, and *UGT74G1*). Moreover, the expression of relative four genes was significantly positively correlated with each other (0.959, 0.989, 0.943, 0.950, 0.928, and 0.957). Conversely, DPPH was negatively significantly correlated with TPC, *ent-KO*, *UGT85C2*, *UGT74G1*, and *UGT76G1* (− 0.588, − 0.728, − 0.678, − 0.681, and − 0.689), respectively.

### Multivariate principal component analysis

**Fig. 8:**
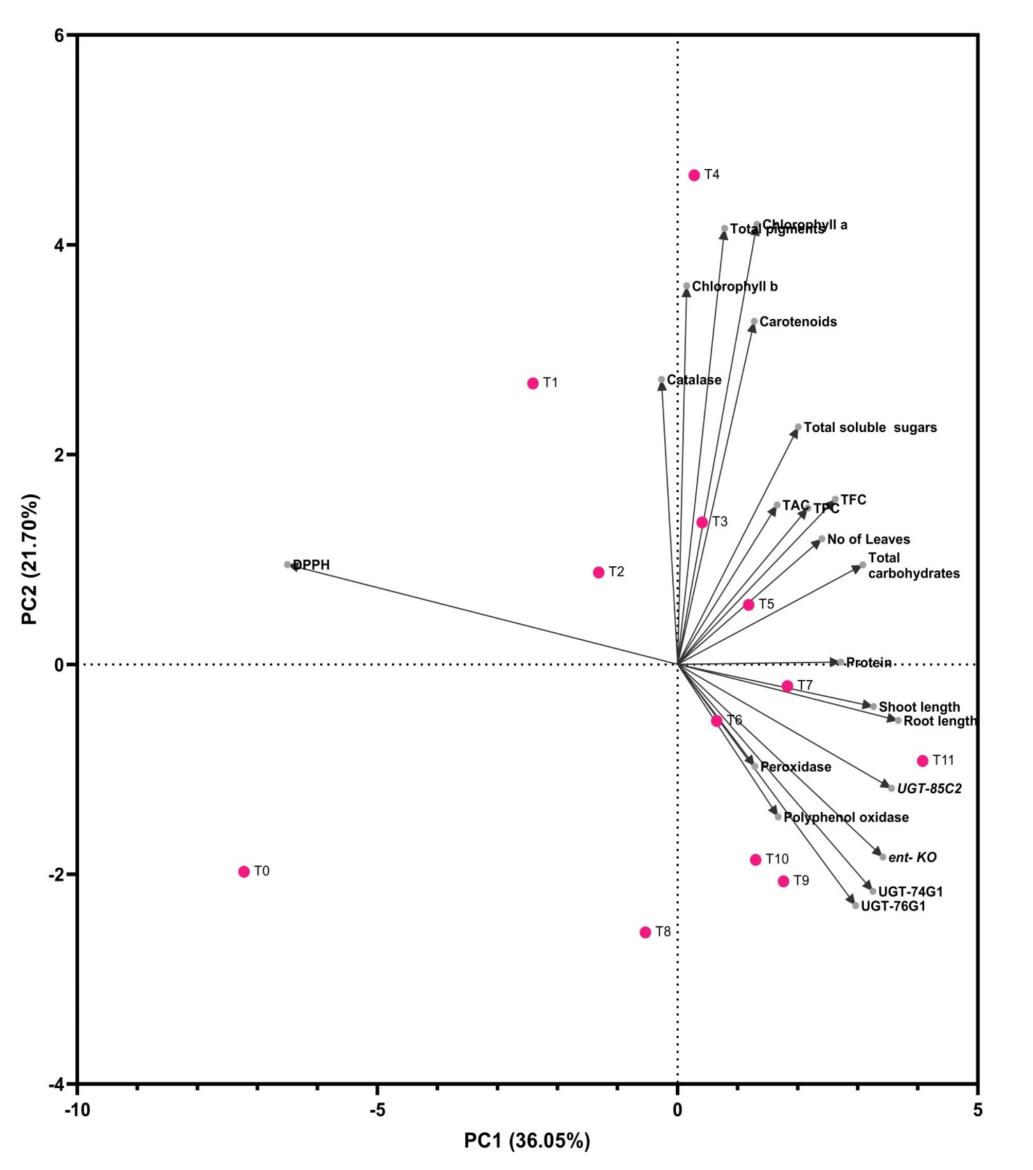
Perceptual mapping (Biplot) analysis showing results of morphological, physiological parameters and expression of steviols glycosides gene. Pink dots enclosed each treatment separated by parameters. TAC, Total antioxidants capacity; TPC, Total phenolics content; TFC, Total flavonoids content.

Principal component analysis (PCA) was performed on all examined parameters, accounting for 57.75% of the total variance in the data set, with the first principal component (PC1) accounting for 36.05% and the second accounting for 21.70% (PC2) as constructed in Fig. 8. PC1 was associated with parameters except for DPPH and CAT and associated with PC2, and treatments were divided into three groups through the vector and cosine. PCA results show that group 1 treated T0, T1, T2, and T8 samples with frequent vectors of DPPH and CAT. Group 2 treated T3, T4 and T5 samples with frequent vectors (chla, chlb, carotenoids, total pigments, number of leaves, carbohydrates TSS, TPC, TFC, TAC and protein). Group 3 treated T6, T7, T9, T10 and T11 with the frequent vectors SHL, RL, POD, PPO, *ent-KO*, *UGT85C2*, *UGT74G1*, and *UGT76G1*. The length of the vector showed the discrimination power of the parameter. In this study, vector of SHL, RL, number of leaves, carbohydrates, protein, CAT, POD, PPO, TPC, TFC, and TAC were of shorter length, which showed poor discrimination power for these traits. On the contrary, the longer vector showed strengthened discrimination of traits such as DPPH, chla, chlb, carotenoids, total pigment, SHL, RL, *ent-KO*, *UGT85C2*, *UGT74G1*, and *UGT76G1*.

## Discussion

Beneficial microorganisms can improve plant nutrient uptake, modify soil health, boost plant defense, and ensure agricultural sustainability^34^. In the same manner, increasing plant length, the number of leaves, and/or SGs content can overcome the scarcity of *S. rebaudiana *production. The primary sources of SGs are leaves, which may include unique endophytic bacteria and provide a better environment for bacteria than other plants used as bio-inoculants^35^. This investigation characterized three endophytic bacterial strains isolated from surface sterilized leaves of *S. rebaudiana* Egy1. The molecular identification illustrated that these isolates belong to the phylum Firmicutes, which have excellent activity of PGP. Thus far, only one metagenomic study of the microbiome in leaves has confirmed our findings. It revealed that Proteobacteria dominated the endophytic bacteria in *S. rebaudiana* leaves, followed by Firmicutes and *Bacillus *species in the core microbiome of leaves^13^.

IAA increases number, length, and volume of adventitious roots, allowing them to transport nutrients^36^. Conversely, GA plays a role in stem elongation, seed dormancy reduction, and flowering^31^. *B. licheniformis* SrAM2 produced the most IAA, as *B. licheniformis* M16 and *B. licheniformis* GL174 were isolated from a corn rhizosphere and *Vitis vinifera *internal tissues^37,38^. Moreover, *B. paralicheniformis* SrAM3 produced the least quantity of IAA, similar to *B. paralicheniformis *TRQ65 from wheat^39^, whereas *B. paramycoides* SrAM4 represents higher levels than typically seen in other bacteria, *B. paramycoides* CSL3, isolated from *Camellia *spp^40^. Similarly, *B. licheniformis* SrAM2 had the maximum GA_3_ production and was higher than *B. licheniformis *DS3 isolated from banana rhizosphere^41^. However, *B. paralicheniformis* promoted plant growth and screened for PGP traits, but there was no evidence about GA_3_ production^42^. *B. paramycoides* SrAM4 production of GA_3_ was the highest between the similar strains isolated from the root of cultivated rice (*Oryza sativa*) and wild rice (*Oryza rufipogon*)^43^.

Nitrogen and phosphate are essential elements for plant growth and development^44^. Despite the availability of nitrogen in the atmosphere as well as phosphate in soil, it is nonetheless unavailable to plants^45^. In this study, the three strains can fix nitrogen and solubilize phosphate, except *B. paramycoides *SrAM4, which was not able to solubilize phosphate. Borah, et al.^40^ did not support our findings; they found that *B. paramycoides* strains could solubilize phosphate. On the other hand, the presence of the *nif* gene in *Bacillus *species^46^. Our results confirmed the fact that most bacteria were able to fix nitrogen and were able to produce ammonia, which provided nitrogen to the host plant, supported plant biomass production, and contributed to plant defense^47^. Furthermore, all isolates produced ammonia, which was consistent with the study proving that *Bacillus *species have the higher ammonia production than other species isolated from chickpea plants^48^. Furthermore, ammonia might be produced by *B. licheniformis* S26 and *B. paramycoides* R5 that were isolated from sugarcane as well as *B. paralicheniformis *AB330 that was isolated from the tea rhizosphere^11^. *Pseudomonas florescences* which has promote plant growth criteria increase *Arachis hypogaea *L. growth as described by Nithyapriya, et al.^49^.

On the other hand, *B. licheniformis* SrAM2 was the only strain able to produce HCN, and the result supported *B. licheniformis* QA1 isolated from *Chenopodium quinoa* and *B. licheniformis* PC-41 isolated from *Zea mays*^50,51^. EPS production is essential for protecting plants from stress and root from desiccation^52^. *Bacillus *appears to have formed biofilms in response to specific environmental changes, including salt stress, nutrients, and oxygen availability^53^. Several studies have shown that *Bacillus*species can produce much more EPS than other species^54^. Additionally, *B. licheniformis* SrAM3 produced higher EPS than *B. licheniformis* B3-15 ^55^.

Since hydrolytic enzymes contribute to organic matter decomposition, protect plants against phytopathogens, and aid bacteria in penetrating plant roots^56^. Hazarika, et al.^8^ investigated the ability of *Bacillus* sp. to produce amylase and protease enzymes but the inability of producing cellulase. *B. licheniformis* SrAM2 and *B. licheniformis *H7 were matched in their ability to produce cellulase only^57^. In this investigation, all enzymes except cellulase were produced by *B. paralicheniformis* SrAM3, consistent with tea rhizobacteria *B. paralicheniformis* AB330 ^11^. *B. paramycoides* SrAM4 produced all of the enzymes found in *B. paramycoides* Vb6, isolated from the rhizosphere soil of *Vicia faba*^58^.

As a result, a better understanding of the relationships between plant-associated bacterial communities is vital in managing crops more effectively. In the present study morphological and physiological traits and expression of SGs genes were lower in non-inoculated plants than inoculated. Our findings confirmed that endophytic bacteria could increase *S. rebaudiana *biomass which was supported by Finkel, et al.^59^. Ye, et al.^60^ study on *S. rebaudiana* inoculated with *Bacillus* endophytic bacteria isolated from *Polygonum hydropiper* showed a significant increase in the number of leaves, SHL, and RL. The increase in plant performance found in dual inoculations and co-inoculations contained *A. brasilense* in corroboration with the effects of *(A) brasilense* and *Bradyrhizobium *spp. co-inoculation on soybean^61^. The number of leaves showed that the highest treatment contains *(B) paralicheniformis* SrAM3 which had the most PGP traits. Similarly, the isolate *B. subtilis *NA-108 increased the number of leaves on strawberries^62^.

In contrast, chlorophyll showed the maximum in plants treated with GA_3,_which was in agreement with other studies of Oat cultivars^63^. Similarly, Datta, et al.^64^ reported that GA_3_treatment boosted chlorophyll and photosynthetic rate in soybean. Modi, et al.^65^ supported our findings as the highest chlorophyll content was observed in 60 mM of GA application. In a comparable manner it was discovered that the foliar application of GA_3_improved plant length, lateral shoot length, lateral shoot number, fruit number, root length, root wet weight, and stem wet weight, total chlorophyll, pigment content, and reducing sugar^66^. Furthermore, chlorophyll content was enhanced and showed maximum after foliar and irrigation with purple phototrophic bacteria (PPB)^67^. It can be due to the ability of bacteria for nitrogen fixation and solubilize phosphate, which is the main element in chlorophyll and photosynthesis^68^. After bacterial inoculation, carotenoids showed improvement in rice as well as alfalfa plants after the application of exogenous hormones compared with control^69^.

As a result, GA_3_ and bacterial treatment played a critical role in boosting carbohydrate and TSS content of plants that is wholly supported with effects of *Phaseolus vulgaris *after treatments with IAA, BA, and endophytic bacteria^70^. TSS were significantly increased after treatments stimulation which was consistent with the content in *S. rebaudiana *treated with PPB^67^. These stimulations may correlate with pigments content as photosynthesis increases, stimulating plant growth and increasing bioactive compounds^26^. Total protein increased in all treated plants mainly co-inoculated, supported with chickpea plant co-inoculated with *B. subtilis* and *Mesorhizobium ciceri* IC53 ^71^. Pandey, et al.^72^ found that dual inoculation of *B. pumilus* and *B. subtilis* significantly boosted different proximate constituents and essential amino acids in Amaranth grains. The increased protein and carbohydrate levels of *Amaranthus hypochondriacus* could be explained that the *Bacillus *isolates facilitated resource acquisition^73^.

A diversity of important microbial genera have been linked to increased secondary metabolite synthesis in medicinal and aromatic plants^74^. All enzymatic and non-enzymatic antioxidants significantly increased in *S. rebaudiana *Shou-2 compared with control; a positive effect of PGPB on TPCs in rice has also been described by Chamam, et al.^75^. Furthermore, our finding agreed with the results of TPCs, TFCs, and DPPH in *S. rebaudiana* due to the combined effect of *Piriformospora indica* and *Azotobacter chroococcum*^76^. In comparison to untreated plants, *Ocimum basilicum *plants treated with microorganisms had considerably greater TPC and TFC^77^. Additionally, the increase of antioxidants may be interpreted by two reasons stimulate the plant’s inducible defense mechanisms in the same way pathogenic microorganisms do as a result of hydrolytic enzymes until the plant adapts to bacterial colonization^78^. Secondly, PGPB may act as priming elicitors which induce these bioactive compounds that formed via the shikimate pathway in higher plants and microorganisms^79^.

We supposed that up-regulation in the antioxidant was associated with the activation of the shikimate pathway may be followed by up-regulation in the MEP pathway, which supported by our results that transcription of SGs pathways genes up-regulated all relative gene expression in treated samples. The common factor between all treatments was GA_3_availability, whether from bacteria secretions or exogenous^80^. The up-regulation of the gene results entirely supported the gene expression results of the same genes in *S. rebaudiana* treated with GA_3_foliar spray^81,82^. The study of Kumar, et al.^83^ supported our results, which showed up-regulation of the gene’s transcription after GA_3_ treatments. Interestingly, the relationship between SGs content and the transcription of some critical genes involved in the SGs production pathway was affected. Our results supported the fact that there was a correlation between *ent-KO *transcription and the accumulation of SGs, which was also confirmed by Kumar, et al.^83^ and our previous study^84^. As the highest stevioside content among the selected samples was compatible with relative gene expression results which supported the study of Ahmad, et al.^33^. It might be described as the uptake of GA_3_ foliar spray producing an excess in GA_3_ in plant cells, which caused the GA_3_biosynthesis pathway to be diverted to SGs biosynthesis^65^. In addition, the increase in stevioside content was supposed to be correlated with carbohydrates and especially TSS content as the glucose units enrichment in SGs chemical structure.

The findings demonstrated a synergistic relationship between the number of leaves, gene expression, and SG production in response to co-inoculations with multiple strains. On the other hand, dual inoculation outperformed single inoculation in terms of plant growth and performance. 

## Materials and methods

### Plant samples and isolation of endophytic bacteria

*S. rebaudiana* Egy1 plantlets obtained from the Sugars Crops Research Institute (SCRI), Agriculture Research Center (ARC), Giza, Egypt. Healthy *S. rebaudiana *Egy1 leaves were randomly collected from different pots, washed under running water, and then surface sterilized according to Zhou, et al.^85^. Leaves were cut into small pieces and plated on Luria–Bertani (LB) agar at 28 °C for 24 h; morphologically distinct colonies were selected, described, and bacteria examined under an oil immersion lens^86,87^.

### Identification of isolates by 16s rRNA

Genomic DNA of bacteria was extracted using SolGent purification kits (SolGent, Daejeon, Korea). After 15 min of incubation at 95 °C, PCR amplified 16s rRNA gene using forward primer (27f) 5′-AGA GTT TGA TCC TGG CTC AG-3′′ and 1492 reverse primer R 5′-GGT TAC CTT GTT ACG ACT T-3′′^88^. PCR conditions were 30 cycles at 95 °C for 20 s, 50 °C for 40 s, 72 °C for 1 min and 30 s, and 72 °C for 5 min. The product was purified using SolGent cleanup kit, checked with 1% agarose gel, and then sequenced on AB1 3730XL DNA Analyzer (ABI, CA, USA). The sequences were identified using the basic local alignment search tool (BLAST) and compared with National Center for Biotechnology Information (NCBI) database (http://www.ncbi.nlm.nih.gov). The phylogenetic relationships were constructed using the neighbor-joining method with 1000 bootstrap replications by Mega X software^89^.

### Screening for plant growth-promoting traits of endophytic bacterial strains

#### Quantitative evaluation of IAA and ga3 production

Isolated strains inoculated in LB broth medium supplemented with 1-tryptophan (1.02 g/L) and incubated in orbital shaking incubator (JSR-100 C/Korea) at 28 °C for 3 days at 150 rpm for IAA determination as Bric, et al.^90^ described. The culture centrifuged for 3 min at 7000 rpm, and the supernatant was used to determine IAA concentrations according to Naveed, et al.^91^.

GA_3_was assessed according to Holbrook, et al.^92^ using LB broth media inoculated with endophytic bacteria and incubated for 7 days at 150 rpm in an orbital shaking incubator, centrifuged at 5000 rpm and then 2 mL zinc acetate reagent was added to 15 mL supernatant. After 2 min, 2 mL 10.6% potassium ferrocyanide was added and centrifuged at 2000 rpm for 15 min. The absorbance was measured at 254 nm (Jenway spectrophotometer model 7315) after adding 5 mL 30% HCl to 5 mL supernatant followed by incubation for 75 min at 25 ºC. GA_3_ concentrations were calculated using GA_3_as a standard in amounts ranging from (100–1000 µg/mL)^93^.

#### Potentiality for nitrogen fixation and phosphate solubilization

N_2_fixation was determined by streaking strains on Jensen’s nitrogen-free medium for 4–7 days at 28ºC^94^, although phosphate capability was detected using Pikovskaya agar medium^95^.

#### Quantitative determination of ammonia, EPS and HCN production

Ammonia was determined according to Cappuccino and Sherman^96^ by inoculating water peptone broth medium with strains, incubated for 4 days in an orbital shaking incubator at 150 rpm at 28 ºC. Ammonia detected using Nessler’s reagent, the development of color ranging from yellow to brown indicated ammonia production and measured at 450 nm.

EPS were determined using phenol sulfuric method and concentrations were calculated by a glucose solution standard^97^. Finally, HCN was assessed by inoculating King’s B agar medium supplemented with 4.4 g/l glycine with bacteria. Filter paper saturated with 0.5% picric acid and 2% sodium carbonate solution was deposited on top of the plate, sealed with parafilm and incubated for 48 h at 28 ºC^98^. A change in color of indicator paper from deep yellow to reddish-brown confirmed production of HCN and the concentration quantitatively assayed according to Reetha, et al.^99^.

#### Extracellular hydrolytic enzymes activities

Hydrolytic enzymes were detected after inoculation of bacteria on agar media containing indicative substrate and incubated at 28 °C for 2–5 days. Amylase activity was detected using iodine solution after streaking of bacteria on a starch agar medium^100^. The cellulase activity was detected according to Khianngam, et al.^101^ using 0.2% aqueous Congo red reagent. Protease activity was observed through a clear zone around colonies inoculated on skimmed milk agar medium described by Cui, et al.^102^. On the other hand, lipase was detected after inoculation on a Tween agar medium^100^.

### Pot experiment using *S. rebaudiana* Shou-2 plantlets

The experiments were conducted at the garden of Faculty of Science, Mansoura University, Dakahlia, Egypt, under 12/12 h light/dark natural light conditions at 28 ± 2 ºC. The plantlets *S. rebaudiana* Shou-2 obtained from SCRI aged 45 days, which were transferred to plastic pots filled with 3.5 kg of soil composed of peat moss, clay, and sand 1:1:1.

A preliminary experiment was conducted to evaluate the synergistic and antagonistic interactions between the strains involved in the microbe bio-formulations, which include of *A. brasilense* ATCC 29,145 obtained from Microbial Resource Center (Cairo Mircen). *Bacillus cereus* SrAM1 MW042692 was previously isolated from *S. rebaudiana*^84^ as well as the three isolated endophytic bacteria (SrAM2, SrAM3, and SrAM4) of this study. GA_3_was prepared in concentration of 60 mM according to Modi, et al.^65^ with modification.

The experiment had 12 treatments that were described in Table 1, applied by foliar spray and irrigation of bacterial suspension every 2 days on 45 days old plantlets for 30 days, while GA_3_ was only foliar sprayed twice with the distance of 2 weeks. The harvesting time was after being treated for 10 days at 85 days old of plant.

Bacterial suspension was prepared by inoculating 10^8^ CFU/mL in 450 mL LB broth, incubated at 28 °C ± 2 °C for 24 h at 150 rpm, centrifuged at 5000 rpm for 15 min, then pellet was resuspended in distilled water and adjusted to OD_600_. Treatments were applied via irrigation of 100 mL bacterial suspension around the root and foliar spray starting with 10 mL at the early stage of the plant and increased until reaching to 25 mL at later stages according to Wu, et al.^67^ with some modifications.

### Assessment of plant growth measurements and pigments

Growth parameters such as number of leaves and shoot and root length. Additionally, chlorophyll a (chl a) and chlorophyll b (chl b) were determined by homogenization of (0.1 g) fresh leaves in 10 mL 80% acetone, the absorbance of homogenate was measured at 470, 645 and 663 nm according to Arnon^103^, whereas carotenoids were according to Lichtenthaler and Wellburn^104^.

### Determination of total soluble sugars (TSS), carbohydrate, and protein content

TSS were determined using the method of Yemm and Willis^105^. Total carbohydrates content was determined according to Hedge, et al.^106^. Finally, protein content in fresh leaves was estimated according to Bradford^107^.

### Measurements of total phenolics (TPC), total flavonoids (TFC), total antioxidants capacity (TAC), and 2,2-diphenyl-1-picrylhydrazyl radical assay (DPPH)

Using methanolic extract, non-enzymatic antioxidants were assessed. The Folin-Ciocalteu method determined TPC of the extracts was expressed as mg gallic acid equivalents (GAE) per gram of dry weight^108^. An aluminum chloride colorimetric test was used to measure TFC and was represented as mg quercetin (QE) equivalents per gram of dry weight^109^. TAC was estimated by phosphomolybdenum assay and expressed as mg ascorbic acid per gram of dry weight^110^. Finally, DPPH was used to assess antioxidant activity by measuring the radical scavenging percentage of the extracts using the following equation^111^.

$$Scavenging\,of\,DPPH\% =\left[ {\left( {A0 - A1} \right)/A0} \right]*100;$$

Where A0 = control absorbance and A1 = extracts absorbance.

### Determination of enzymatic antioxidant activity

For enzymes assays, the extract was prepared by macerating 0.2 g of fresh leaves in a cold 3 mL (0.05 M) phosphate buffer (pH = 7), the homogenate was filtrated, and the filtrate was made up to 5 mL. Using the method provided by Aebi^112^, the catalase (CAT) activity was determined. In contrast, the polyphenol oxidase (PPO) and peroxidase (POD) activity assay were determined using the method reported by Kar and Mishra^113^.

### Real-time-qPCR analysis of transcript levels of specific genes associated with the biosynthesis of SGs

Total RNA was extracted from frozen leaves using the RNeasy Plant Mini kit (Qiagen, Hilden,Germany); the first-strand cDNA was generated with the Qiagen Reverse Transcription kit. The QuantiTect SYBR Green PCR kit technique was used to perform RT-qPCR; SYBR green binds to double-stranded DNA, and the amplification products quantify the development of the target transcript compared with the housekeeping gene transcript. RT-qPCR was performed in a 96-well plate in a total volume of 10 µL, with 1.5 µL of cDNA (4 ng/µL), 0.1 µL of each primer (10 pm/µL), and 5 µL SYBER Green qPCR master mix (Qiagen, Hilden,Germany). PCR amplification conditions were 95 °C for 20 s, followed by 40 cycles of 95 °C for 30 s, 62 °C for 30 s, and 72 °C for 1 min, followed by a melting cycle from 65 °C to 95 °C to validate amplicon specificity. Amplification was performed using Hajihashemi and Geuns^114^ gene-specific primers (Supplementary Table 1). In this investigation, the housekeeping gene *β-Actin* was used, and the relative expression levels were measured using a 2^−ΔΔCt^assay^115^.

### High-performance liquid chromatography analysis of stevioside

Twenty mg of dried leaves was extracted three times by boiling for 15 min in 3 mL deionized water. The extracts were centrifuged, and the supernatant was decanted, cooled, and made up to 15 mL with deionized water. According to Ceunen, et al.^116^, the quantity of stevioside was determined. In the high-performance liquid chromatography (HPLC, Water 2960 Alliance HPLC system), 100 µL of the extract was injected in C18 columns Xterra: 4.6 × 100 mm, 5 μm; UV detection was 210 nm. The solvent flow rate was 1 mL/min, and the acetonitrile: 1.0 mM phosphoric acid gradient.

### Statistical analysis

All experiments used a randomized complete block design with three biological and technical experimental replications. Statistical calculations were carried out with SPSS (version 16; SPSS, New York, NY, USA). The results were presented as mean values with standard errors (±). The mean values were subjected to one-way analysis of variance ANOVA and Duncan’s multiple range tests^117^. Values of *p* ≤ 0.05 were considered as significant using superscripted letters. Perceptual mapping (Biplot) analysis, heatmap of simple linear Pearson correlation coefficient between parameters and histograms were plotted using Sigma Plot 14 software and GraphPad Prism9 (GraphPad Software, Inc.).

## Conclusions

*S. rebaudiana* endophytic bacteria demonstrated potency for plant growth promotion in single, dual, or mixed bio-formulations. As a result, PGPB tends to play an important role in the development of plant vigour and health by delivering vital nutrients, bioactive substances, and gene expression in *S. rebaudiana* Shou-2. When compared to control, the treatments increased all parameters, but co-inoculations were the most effective on stevioside content, which was supported by an elevation in the expression of SGs related genes. Furthermore, an increase in carbohydrate and TSS contents was thought to be associated with the elevated in SGs, particularly the stevioside content. The activation of the shikimate pathway is associated with increases in antioxidant content, which is thought to be an indication of MEP pathway activation. From the foregoing, several characteristics produced by endophytic bacteria and exogenously applied strains work together to improve plant performance and the development of promising bioactive compounds.

## Electronic supplementary material

Below is the link to the electronic supplementary material.

Supplementary Material 1

## Data Availability

All data needed to evaluate the conclusions in the paper are presented in the paper and/or the supplementary materials.

## Abbreviations

CAT: Catalase
Chla: Chlorophyll a
Chlb: Chlorophyll b
DPPH: 2,2-Diphenyl-1-picrylhydrazyl
EPS: Exopolysaccharides
GA: Gibberellic acid
GAE: Gallic acid
HCN: Hydrogen cyanide
HPLC: High-Performance Liquid Chromatography
IAA: Indole-3-acetic acid
LB: Luria–Bertani
MEP: C-methyl-D-erythritol 4-phosphate
PGP: Plant growth promoting
PGPB: Plant growth-promoting bacteria
PGPR: Plant growth promoting rhizobacteria
POD: Peroxidase
PPO: Polyphenol oxidase
QE: Quercetin
RL: Root length
SE: Standard error
SGs: Steviol glycosides
SHL: Shoot length
TAC: Total antioxidants capacity
TFC: Total flavonoid content
TPC: Total phenolic content
TSS: Total soluble sugars
UGTs: UDP glycosyltransferases

## Declarations

### Plant material permission

All authors state appropriate permissions and/or licenses for collection of plant or seed specimens used in this study.
