# Supplementary material for: Comparison between bacterial bio-formulations and gibberellic acid effects on Stevia rebaudiana growth and production of steviol glycosides through regulating their encoding genes
Source: Sci Rep. 2024 Oct 15;14:24130. doi: 10.1038/s41598-024-73470-0 (PMC11480349; doi:10.1038/s41598-024-73470-0)
Supplement: Supplementary file 1 — Supplementary Material 1 [file 41598_2024_73470_MOESM1_ESM.docx]

**Supplementary Table 1.** List of genes and designed primers and house-keeping genes used in RT-qPCR. *Ent-KO,* *UGT85C2*, *UGT74G1* and *UGT76G1* as well as *β-Actin* (Hajihashemi and Geuns 2016).

| **Gene** | **Primer sequence 5'->3' (Forward/Reverse)** | **Amplicon length (bp)** | **Accession number** |
| --- | --- | --- | --- |
| ***ent-KO*** | F: GCTGTGATGAAGTCTCTTATTAAA/  R: CCATAGTGGTGTCTGATGATTCAAT | 162 | AY364317 |
| ***UGT85C2*** | F: TCGATGAGTTGGAGCCTAGTATT/  R: CTAAACTGTATCCATGGAGACTC | 153 | AY345978 |
| ***UGT74G1*** | F: TGCATGAACTGGTTAGACGATAAG/  R: GCATCCTACTGATTCGTGTGCTA | 274 | AY345982 |
| ***UGT76G1*** | F: GCAGCTTACTAGACCACGATC/  R: CTCATCCACTTCACTAGTACTAC | 107 | AY345974 |
| ***β-Actin*** | F: AGCAACTGGGATGACATGGAA/  R: GGAGCGACACGAAGTTCATTG | 65 | AF548026 |

**Supplementary Table 2.** Pearson correlation between growth parameters, biochemical features and the expression of genes involved in steviols biosynthesis of *S. rebaudiana* Shou-2 shoot system.

|  | SHL | RL | No of leaves | Chl a | Chl b | Carotenoids | TP | TSS | Carb. | Protein | TPC | TFC | TAC | DPPH | CAT | POD | PPO | ent-KO | UGT-85C2 | UGT-74G1 | UGT-76G1 |
| --- | --- | --- | --- | --- | --- | --- | --- | --- | --- | --- | --- | --- | --- | --- | --- | --- | --- | --- | --- | --- | --- |
| SHL | 1.000 |  |  |  |  |  |  |  |  |  |  |  |  |  |  |  |  |  |  |  |  |
| RL | 0.923* | 1.000 |  |  |  |  |  |  |  |  |  |  |  |  |  |  |  |  |  |  |  |
| No of leaves | 0.386 | 0.394 | 1.000 |  |  |  |  |  |  |  |  |  |  |  |  |  |  |  |  |  |  |
| Chl a | 0.220 | 0.216 | 0.373 | 1.000 |  |  |  |  |  |  |  |  |  |  |  |  |  |  |  |  |  |
| Chl b | 0.041 | -0.056 | 0.160 | 0.816* | 1.000 |  |  |  |  |  |  |  |  |  |  |  |  |  |  |  |  |
| Carotenoids | 0.112 | 0.210 | 0.277 | 0.759* | 0.298 | 1.000 |  |  |  |  |  |  |  |  |  |  |  |  |  |  |  |
| TP | 0.129 | 0.084 | 0.278 | 0.964* | 0.938* | 0.599* | 1.000 |  |  |  |  |  |  |  |  |  |  |  |  |  |  |
| TSS | 0.266 | 0.382 | 0.403 | 0.486 | 0.171 | 0.617* | 0.370 | 1.000 |  |  |  |  |  |  |  |  |  |  |  |  |  |
| Carb. | 0.654* | 0.800* | 0.114 | 0.458 | 0.173 | 0.500 | 0.346 | 0.568 | 1.000 |  |  |  |  |  |  |  |  |  |  |  |  |
| Protein | 0.393 | 0.509 | 0.674* | 0.208 | -0.192 | 0.425 | 0.028 | 0.258 | 0.390 | 1.000 |  |  |  |  |  |  |  |  |  |  |  |
| TPC | 0.492 | 0.508 | 0.339 | 0.405 | 0.348 | 0.101 | 0.378 | 0.491 | 0.477 | 0.182 | 1.000 |  |  |  |  |  |  |  |  |  |  |
| TFC | 0.641* | 0.550 | 0.737* | 0.489 | 0.434 | 0.255 | 0.478 | 0.528 | 0.404 | 0.316 | 0.458 | 1.000 |  |  |  |  |  |  |  |  |  |
| TAC | 0.209 | 0.269 | 0.487 | 0.407 | 0.117 | 0.402 | 0.283 | 0.144 | 0.335 | 0.589* | 0.223 | 0.246 | 1.000 |  |  |  |  |  |  |  |  |
| DPPH | -0.525 | -0.575 | -0.548 | -0.039 | 0.016 | 0.101 | 0.003 | -0.298 | -0.341 | -0.57 | -0.589 | -0.447 | -0.102 | 1.000 |  |  |  |  |  |  |  |
| CAT | -0.275 | -0.090 | 0.099 | 0.489 | 0.103 | 0.678* | 0.340 | 0.427 | 0.124 | 0.121 | 0.174 | -0.198 | 0.350 | 0.252 | 1.000 |  |  |  |  |  |  |
| POD | 0.363 | 0.391 | 0.115 | -0.021 | -0.266 | 0.261 | -0.129 | -0.196 | 0.254 | 0.515 | -0.108 | 0.099 | 0.141 | -0.042 | -0.101 | 1.000 |  |  |  |  |  |
| PPO | 0.515 | 0.608* | 0.219 | -0.143 | -0.356 | -0.088 | -0.270 | -0.169 | 0.256 | 0.396 | 0.303 | 0.042 | 0.237 | -0.208 | 0.059 | 0.592* | 1.000 |  |  |  |  |
| ent-KO | 0.620* | 0.779* | 0.319 | -0.069 | -0.272 | 0.043 | -0.176 | 0.345 | 0.622* | 0.484 | 0.303 | 0.361 | 0.162 | -0.728* | -0.167 | 0.204 | 0.292 | 1.000 |  |  |  |
| UGT-85C2 | 0.660* | 0.791* | 0.394 | 0.111 | -0.124 | 0.176 | -0.003 | 0.302 | 0.660* | 0.564 | 0.240 | 0.420 | 0.323 | -0.678* | -0.128 | 0.243 | 0.257 | 0.958* | 1.000 |  |  |
| UGT-74G1 | 0.631* | 0.774* | 0.243 | -0.122 | -0.277 | -0.033 | -0.210 | 0.249 | 0.594* | 0.404 | 0.232 | 0.328 | 0.083 | -0.681* | -0.251 | 0.217 | 0.301 | 0.989* | 0.954* | 1.000 |  |
| UGT-76G1 | 0.496 | 0.644* | 0.298 | -0.163 | -0.230 | -0.144 | -0.214 | 0.124 | 0.464 | 0.415 | 0.125 | 0.306 | 0.143 | -0.688* | -0.333 | 0.127 | 0.207 | 0.942* | 0.928* | 0.957* | 1.000 |

*Flag for significance correlation, significant correlations (P < 0.05),

Where TP, Total pigments and Carbs, carbohydrates.

A
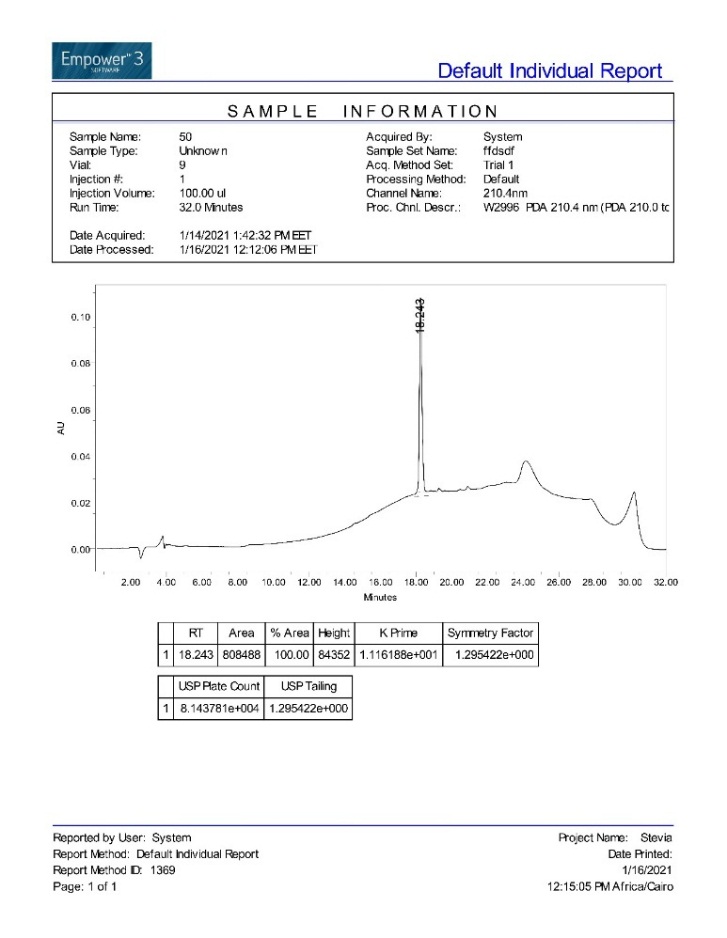
B
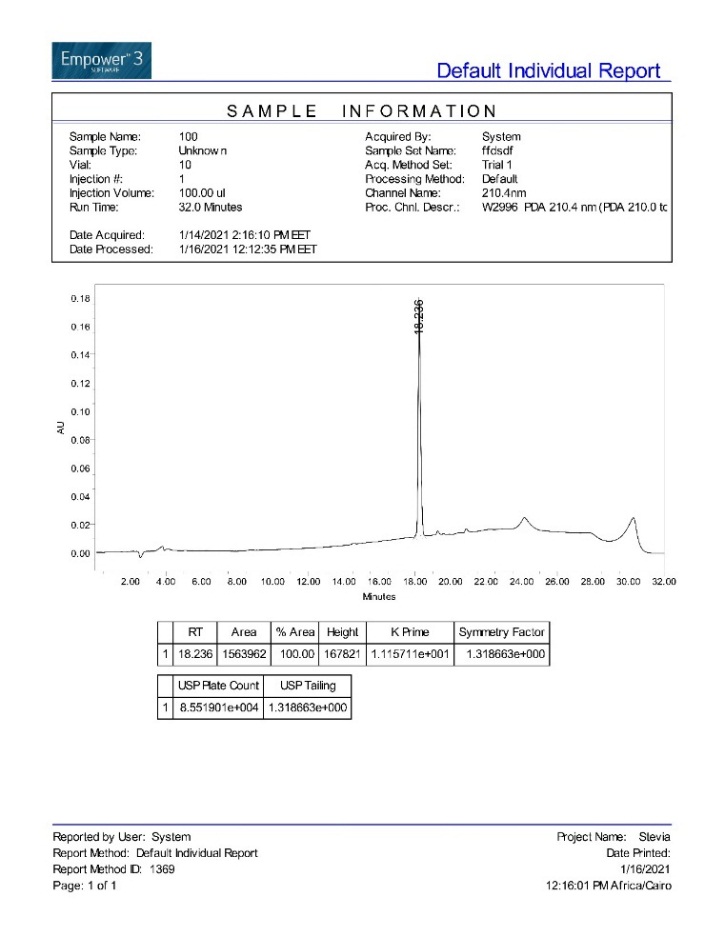


C
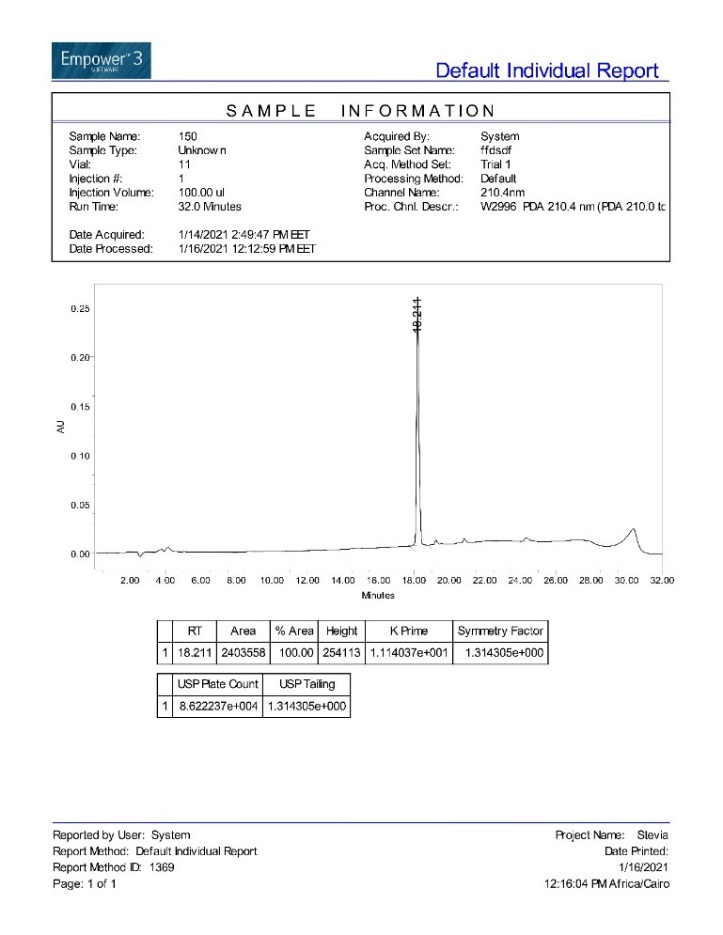
D
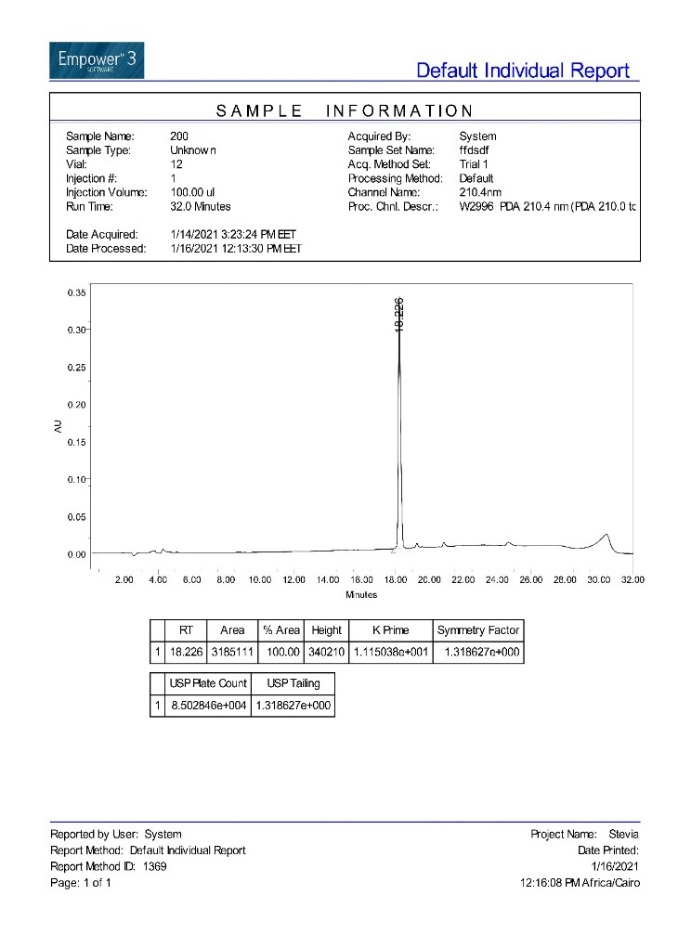


E
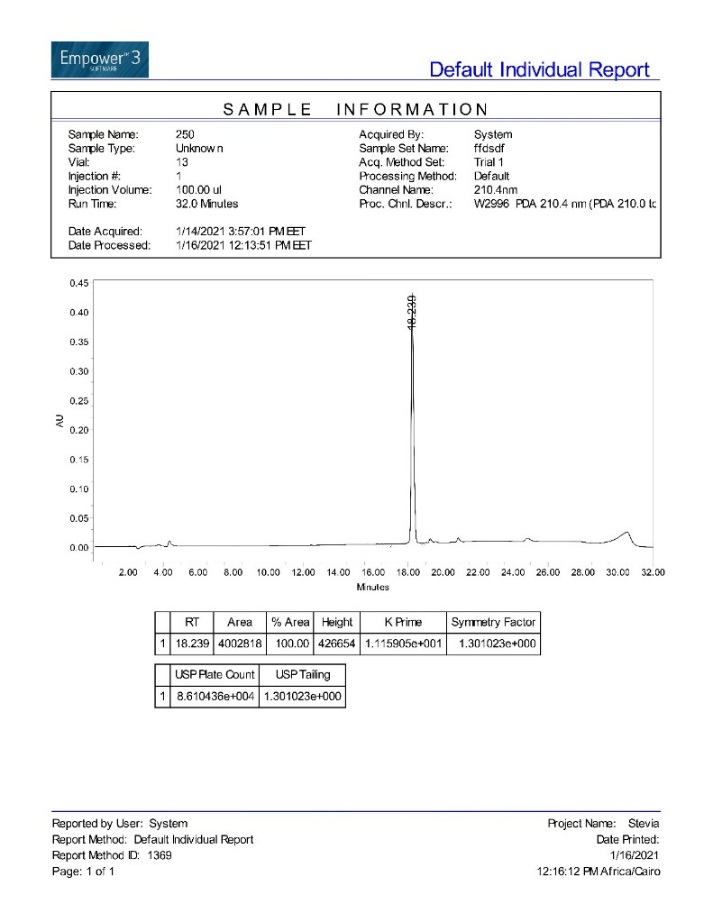
F
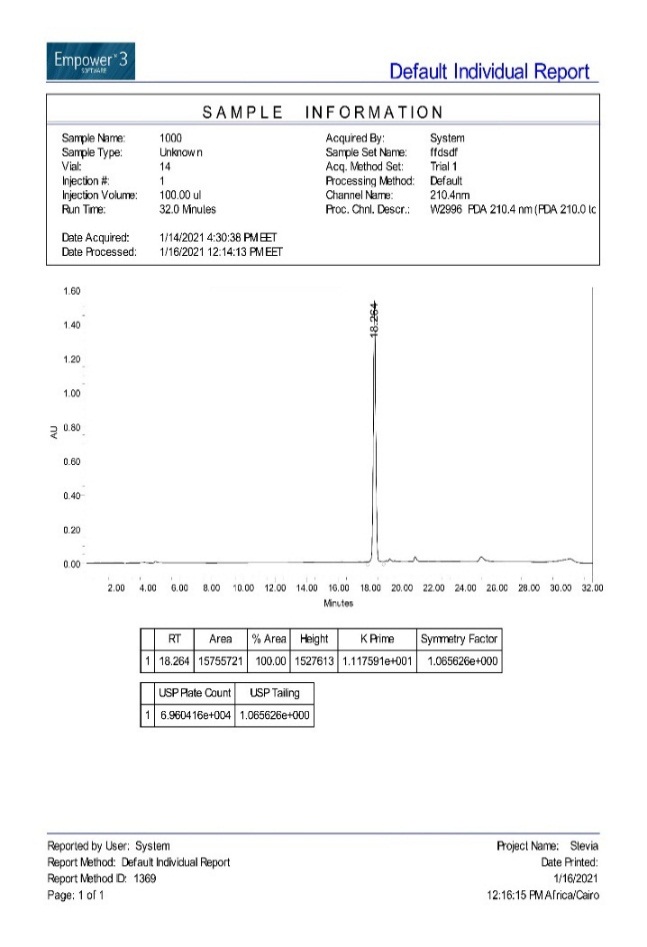


**Supplementary Figure 1.** Chromatogram of different concentrations of standard stevioside used for preparation of standard curve to calculate unknown stevioside content. (A), (B), (C), (D), (E) and (F) concentrations at 50,100,150, 200, 250 and 1000 µg/ml, respectively.

**A
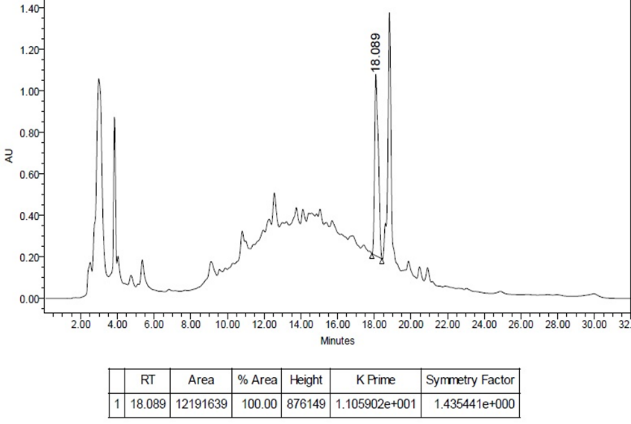
B
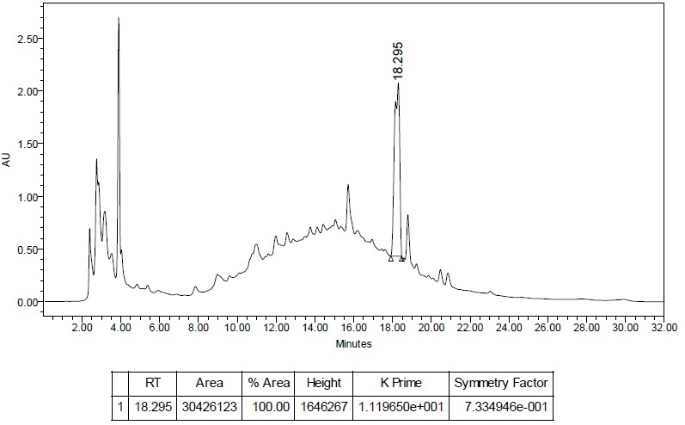
**

**C
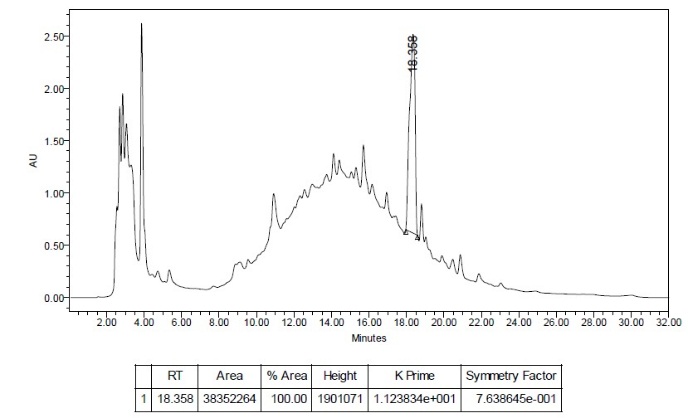
D
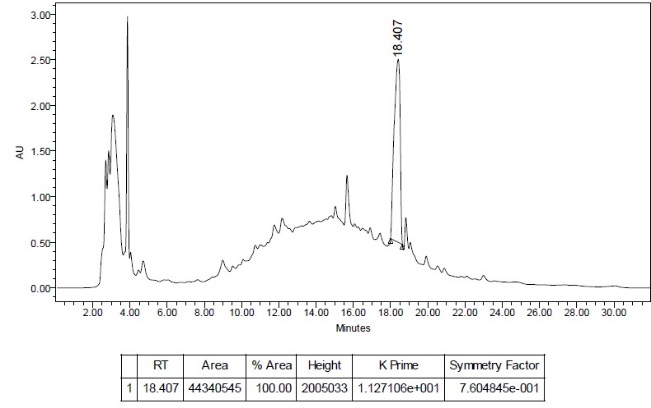
**

**E
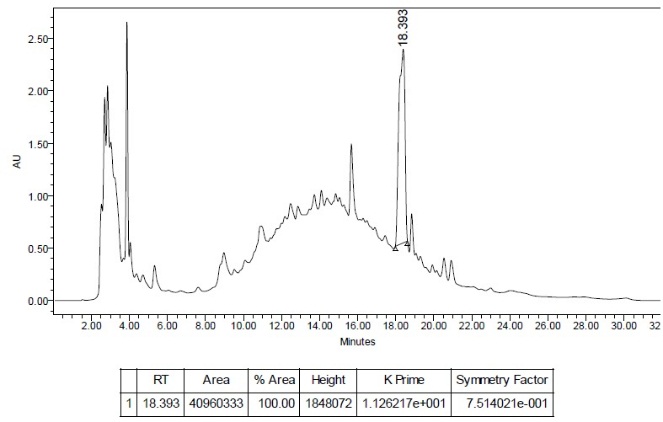
F
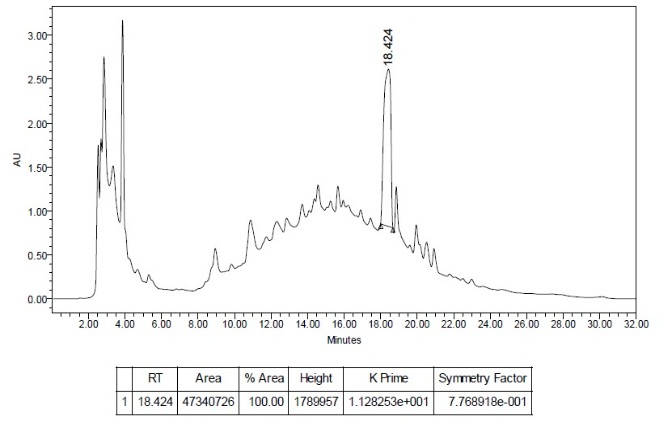
**

**Supplementary Fig.2.** Chromatograms of stevioside content of different treatments **(A)** T0 **(B)** T1 **(C)** T3 **(D)** T6 **(E)** T7 and **(F)** T10.
